# Caring for carers of people with advanced cancer at hospital discharge (CARENET): A single-arm open label feasibility trial

**DOI:** 10.1017/S1478951525100710

**Published:** 2025-09-05

**Authors:** Celia Marston, Marc L'etang, Jennifer Philip, Deidre D Morgan, Lara Edbrooke, Sungwon Chang, Meera R Agar

**Affiliations:** 1 Department of Occupational Therapy, Peter MacCallum Cancer Centre, Melbourne, VIC, Australia; 2 IMPACCT, Faculty of Health, University of Technology Sydney, Sydney, NSW, Australia; 3 Department of Occupational Therapy, Royal Melbourne Hospital, Melbourne VIC, Australia; 4 Department of Social Work, Peter MacCallum Cancer Centre, Melbourne, VIC, Australia; 5 Department of Medicine, University of Melbourne, Melbourne, VIC, Australia; 6 Palliative Care Service, St Vincent’s Hospital, Melbourne, VIC, Australia; 7 Parkville Integrated Palliative Care Service, Peter MacCallum Cancer Centre and Royal Melbourne Hospital, Melbourne, VIC, Australia; 8 Research Centre for Palliative Care, Death and Dying (RePaDD), Palliative and End-of-Life Care, Flinders University, Bedford Park, SA, Australia; 9 Department of Physiotherapy, The University of Melbourne, Melbourne, VIC, Australia; 10 Department of Health Services Research, Peter MacCallum Cancer Centre, Melbourne, VIC, Australia; 11 Sir Peter MacCallum Department of Oncology, The University of Melbourne, Melbourne, VIC, Australia

**Keywords:** Advanced cancer, carers, discharge planning, palliative care

## Abstract

**Objectives:**

Carers are critical to support discharge home from hospital at end of life yet remain under-represented in health service initiatives to assist this transition. A carer-focused intervention embedded into practice may facilitate hospital discharge. This open-labeled, single-arm phase 2 study aimed to determine the feasibility of (1) delivering a multi-staged intervention (CARENET) to carers of advanced cancer patients in a hospital setting and (2) the study design to inform a phase 3 trial.

**Methods:**

CARENET, delivered before and after discharge to address carer support needs, was tested in an Australian specialist cancer hospital. Eligible participants included carers of advanced cancer inpatients with planned discharge home. The primary outcome was intervention and trial feasibility (recruitment and adherence). Secondary outcomes were eligibility and effects of intervention on outcomes including carer preparedness.

**Results:**

Of the 382 potential patient–carer dyads, 25 were recruited within required time frames. The intervention adherence outcome feasibility threshold of 80% of carer participants completing all 3 core components of CARENET was not achieved (60% completion). Trends in improvement in overall carer levels of preparedness were observed from baseline to discharge home (*n* = 12; mean [95% CI]) 0.5 [−0.0007, 1.007]). However, a downward trend in preparedness to provide emotional care after discharge was observed (*n* = 12; mean [95% CI] 0.25 [−0.30, 0.80]).

**Significance of results:**

Delivering all elements of the CARENET intervention to address carers’ needs in the discharge planning context was not feasible. However, some elements were feasible, including identifying and responding to carer need, whilst completing elements after discharge were less feasible. Findings can be explained by problems with adherence, eligibility, and clinician barriers to fitting a multi-staged carer intervention into an acute healthcare setting. Future research should test a more adaptable intervention and delivery model that is accessible to all carers across and compatible with acute care settings.

## Introduction

Moving from hospital to home for people with advanced cancer can be a time of heightened stress and uncertainty for both patients and their carers, where most of the care responsibilities shift onto family or friends (Adejoh et al. 2021). This transition of care is associated with uncontrolled symptoms for the patient and excessive burden on carers who are frequently unaware of what to expect in taking on the caring role for a person with advanced cancer (Lawson et al. 2006). People with advanced cancer will experience hospital-to-home transitions across their illness trajectory, and these are particularly burdensome in the year after diagnosis (Whitney et al. 2017). Some authors have highlighted that symptom burden, disease progression, and carers reaching capacity can lead to increased hospitalization rates (Lawson et al. 2006; Lage et al. 2018).

Carers are critical to patients having a successful discharge from hospital to home at the end of life (Laugaland et al. 2012; Higginson et al. 2013). First, they provide most of the care when a person returns home from hospital, which often incurs an emotional, physical, and financial cost (Wang et al. 2018; Adejoh et al. 2021). Secondly, their capacity to provide care remains one of the main factors that influences a person’s ability to remain at home and avoid readmission to hospital (Laugaland et al. 2012; Higginson et al. 2013). Thirdly, they have a set of unique needs that are different from the patient and routinely remain unmet. These needs include detailed communication and information provision related to support and practical aspects of caring (Harding et al. 2012; Wang et al. 2018).

International policy recommends that carers are included in discharge planning models and their support needs are addressed across the continuum of care (National Institute of Health and Clinical Excellence (NICE) 2004; World Health Organization (WHO) 2016; Naylor et al. 2017; Carers 2021). However, carers of people with advanced disease are under-represented in interventions and service initiatives aimed at improving hospital discharge. A recent systematic review of the reporting quality of hospital-to-home transition intervention studies found that only 22% of studies included carers in intervention design, delivery, and outcomes, and even less so for people with advanced cancer (Marston et al. 2022).

The Carer Support Needs Assessment Tool- Intervention (CSNAT-I) has shown promise as a potential intervention model that could be delivered in the hospital discharge setting (Aoun et al. 2015; Ewing et al. 2018; Hall et al. 2020). Originally developed for and validated with carers of people with advanced cancer in the community palliative care setting (Ewing et al. 2013), the CSNAT-I is a systematic 5-stage process that creates opportunities for addressing carers’ needs and has been demonstrated to improve their ability to cope with care at home (Courvoisier et al. 2015; Toye et al. 2016; Grande et al. 2017). These improvements include a significant reduction in prolonged grief (Grande et al. 2017), carer strain (Courvoisier et al. 2015), and sustained increased preparedness and quality of life (Toye et al. 2016).

Carers and clinicians in a UK study recommended that CSNAT-I be implemented in hospital settings to expand the discharge planning focus to include carers. This could address “unrealistic expectations” of what care at home would entail and may reduce the likelihood of breakdown of care at home after discharge (Ewing et al. 2018). Carers in this study also recommended implementing this tool as early as possible during hospital admission, with a review upon the initial transition home(Ewing et al. 2018). Recent evaluation of the CSNAT-I implemented by palliative care nursing staff in a UK hospital revealed behavioral and organizational challenges impacting its delivery (Hall et al. 2020). The structure and focus of the CSNAT-I were considered acceptable; however, there was uncertainty regarding the “fit” of carers’ needs in the health systems and which health professional should be responsible for delivery of the CSNAT-I (Hall et al. 2020).

To investigate this further, we examined the CSNAT-I in an Australian inpatient acute cancer setting as part of routine care by occupational therapists (OTs; Marston et al. 2023). OTs play an important role in facilitating discharge home at end of life through interventions that optimize patients’ functional performance and carers’ capacity to care (Marston et al. 2015; Eva and Morgan 2018) The CSNAT-I was accepted by patients, carers, and clinicians as an integrated part of existing discharge planning that should reach across hospital and home and target practical needs of caring (Marston et al. 2023). Study findings informed the development of a modified delivery model for the CSNAT-I – **Car**ing for car**e**rs of people with adva**N**c**e**d cancer at hospi**T**al discharge (CARENET). The CARENET model was then used to proceed to the next phase of feasibility testing in a controlled trial. Feasibility refers to the practicality of an intervention – whether it is fit for purpose in a specific context (Bowen et al. 2009; Klaic et al. 2022). Three key areas of feasibility that were assessed were practicality, implementation, and limited efficacy based on Bowen et al.’s (2009) framework. Practicality refers to the extent an intervention can be delivered within the given resources and circumstances. Implementation refers to the extent an intervention can be successfully delivered as intended within a defined context or according to protocol (i.e., fidelity). Limited efficacy refers to the intended effects of an intervention on relevant outcomes (Bowen et al. 2009). Thus, this study aimed to determine the feasibility of (1) delivering the CARENET intervention to carers of advanced cancer patients in an acute oncology setting as intended and (2) the study design to inform a phase 3 trial.

## Methods

### Design

A single-arm open-label study design was employed to determine the feasibility of the CARENET intervention and the study design. The selected study design aligns with the feasibility and piloting stage recommended in the Medical Research Council (MRC) framework for designing and evaluating complex interventions (Skivington et al. 2021). Feasibility was assessed using 3 key areas of focus recommended by Bowen’s framework for feasibility studies (Bowen et al. 2009) – practicality, implementation (fidelity), and limited efficacy, with links of areas of focus to trial aims and measures detailed in Table 1. Quantitative data were collected prospectively to address trial aims. CONSORT guidelines were used for reporting structure (Eldridge et al. 2016).

**Table 1.** Feasibility outcomes and alignment with research questions

Research questions	Areas of focus for feasibility (Bowen et al. 2009)	Components	Endpoints/measures
Primary outcomes			
Can participants be recruited to the CARENET trial within the required timeframe?	Practicality	Recruitment	20 participants enrolled in study within 9 months
Can CARENET and its critical elements be delivered to intended carer participants in a controlled setting?	Implementation	Intervention adherence	At least 80% of enrolled carer participants would have had all 3 aspects of intervention delivered (needs assessment, responding to needs, and after discharge review)
Secondary outcomes			
To what extent can CARENET be successfully carried out with intended carer participants using existing means and resources?	Practicality	Eligibility, attrition/dropouts	Recruitment rate and eligibility data (including reasons for non-recruitment and withdrawal) via screening log and project-specific electronic Case Report Form (eCRF)
Does CARENET show promise of improving outcomes for carers in an acute oncology context?	Limited efficacy/practicality	Completion rates of measures. Intended effects of intervention on outcomes	Service and process data via project-specific eCRF Variances in patient- and carer-related measures^a^ (to power for larger study). Patient measures: AKPS, EQ-5D-5L, RUG-ADL, and SAS; carer measures: Carer Preparedness Scale and CarerQoL-7D

AKPS = Australian Karnofsky Performance Scale; RUG-ADL = Resource Utilization Groups – Activities of Daily Living; EQ-5D-5L = EuroQol 5-Dimensions-5-Levels; SAS = Symptom Assessment Scale; CarerQoL-7D = Carer Quality of Life (QOL) Instrument.

a Supplementary File 2 for measurement properties.

### Participants and setting

Participants were adult inpatients with advanced cancer and their carers (patient–carer dyad). The setting was a 30-bed medical oncology ward situated in a 140-bed specialist cancer center in metropolitan Melbourne. For the purposes of this study, carers were defined according to the NICE definition: “Lay people in a close supportive role who share in the illness experience of the patient and who undertake vital care work and emotion management” (National Institute of Health and Clinical Excellence (NICE) 2004). Advanced cancer was defined as cancer that had spread or returned (recurred) after treatment, and included locally advanced and metastatic cancer. All people with advanced cancer (regardless of prognosis) and identified carers were potentially eligible and screened for the study. Eligibility criteria included: English speaking, identified as a carer of a person with a diagnosis of advanced cancer with a planned discharge home and able to provide informed consent. Planned discharge home was defined as a decision for future discharge supported by an assessment using locally developed criteria to identify a relevant pathway home. Participants were excluded if patients had a planned discharge to residential care or subacute settings. Patients who were part of the dyad and were able to provide informed consent, or had proxy written consent, were eligible and invited to participate if interested.

### Procedure

The study protocol was prospectively registered with the Australian New Zealand Clinical Trials Registry Registration No: ACTRN12622001325796. Ethical approval was obtained by the Peter MacCallum Cancer Centre Ethics Committee (HREC No. 83438 and the UTS Ethics Committee (ETH22-7440). From November 2022 to July 2023, potential participants were identified and referred for eligibility screening. Potentially eligible participants were identified by their own clinicians (medical, nursing, or allied health) and referred to the research assistant (RA) after discussions with potential participants. Eligible participants were invited to partake in the study followed by a formal consent process. Carers were consented first, then if they consented, the patient consent process would occur. The proposed sample size was 20–30 patient–carer dyad participants as per “general rule of thumb” for feasibility studies (Totton et al. 2023) and based on pragmatic findings from the preliminary study.

**Figure 1.**
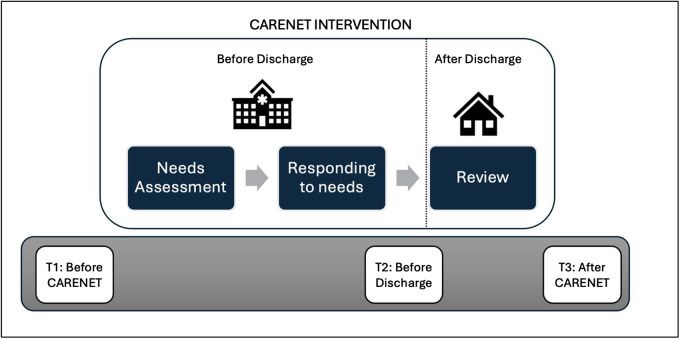
Intervention components (Needs Assessment, Responding to Needs, and Review) and data collection timepoints (T1 – within 1–3 days before CARENET, T2 – within 1–2 days before discharge, and T3 – after CARENET and within 1 week after discharge).

Once consent was obtained, the study involved the collection of measures across 3 time points and the delivery of CARENET across hospital and home settings (before and after hospital discharge). The 3 time points for study measures (T1, T2, and T3) are defined and represented in Figure 1 with details related to the intervention as follows.

### Intervention

The features of the CARENET intervention are described in Supplementary File 1 and represented in Figure 1. CARENET consisted of the CSNAT-I, and a modified intervention delivery mode whereby the CSNAT-I was delivered by OTs as an integrated part of usual discharge planning practices across hospital and home settings. During the hospital stay, carers completed 3 components: (1) an assessment and prioritization of their needs using the paper-based CSNAT-I tool (Needs assessment), (2) an action plan to address their needs (Responding to needs) via support directly from treating OTs or signposting other avenues of support for the carer to initiative themselves, and (3) within 72 h after returning home from hospital, the carers received a review of their needs via a phone call (Review). The assessment part of the CSNAT-I that was embedded in the first component consisted of 15 domains of support need for the carer to choose from. They related to the practical aspects of caring as well as their own personal needs as a carer (Ewing et al. 2013). *The time it took to deliver the CARENET was tailored to the individual and the period the patient spent moving from hospital to home.*

#### Implementation process

OTs completed training and participated in a planned implementation strategy to support the delivery of the intervention. This included a 90-min individual online learning module on the CSNAT-I (2022) and 2 one-hourly group education sessions on the CARENET intervention protocol and trial processes, before recruitment commenced. During recruitment, clinician support and monitoring of adherence occurred via (1) Assignment of OT clinical champions; (2) Expert working group of OTs, researchers and consumers; and (3) File audit and informal assessment of clinician compliance (conducted by RA [M.L.T.] and primary investigator [C.M.]; Supplementary File 3).

### Outcomes

The primary outcome was intervention and trial feasibility (recruitment and adherence). Secondary outcomes were eligibility/attrition (practicality) and intended effect of intervention on patient and carer outcomes (limited efficacy). Alignment of these outcomes with research questions, areas of feasibility, measures, and endpoints are presented in Table 1.

### Data collection

Measures used for data collection are presented in Table 1. Patient- and carer-related data were collected via standardized measures and questionnaires. Service, process data were collected via medical records and project-specific case report forms. As illustrated in Figure 1, data collection at the 3 time points (T1, T2, and T3) for study measures was as follows: T1 1–3 days before intervention commenced; T2 1–2 days before discharge, and T3 after CARENET and within 1 week after discharge. Patient and carer outcome measures were the only measures collected across all 3 time points. Patient-related measures were the Australian Karnofsky Performance Scale (AKPS) (Abernethy et al. 2005), Resource Utilization Groups – Activities of Daily Living (RUG-ADL) (Fries et al. 1994), Symptom Assessment Scale (SAS) (Daveson et al. 2021), and EuroQol 5-Dimensions-5-Levels (EQ-5D-5L) (Herdman et al. 2011). Carer-related measures were Preparedness for Caregiving Scale (Archbold et al. 1990) and Carer Quality of Life (QOL) Instrument (CarerQOL-7D) (Hoefman et al. 2017). The main carer-related outcome was carer-preparedness measured via Preparedness for Caregiving Scale (Archbold et al. 1990), where a high score reflects improved preparedness, which was the only measure collected across all 3 time points. Features of these valid and reliable measures are summarized elsewhere (Supplementary File 2). Demographic data were collected at baseline (T1), while adherence, process, and service data were collected at T3.

### Data analysis

Data analysis was conducted using Excel (Microsoft Office V23.08) and IBM SPSS v28 software. Mean and standard deviation were reported for continuous normally distributed data, and interquartile range and median for data deviating from normal distribution. Results of the categorical variables (eligibility, recruitment, attrition, and intervention adherence) were presented as percentages and frequencies. All questionnaires were scored according to their respective scoring guidelines. To evaluate changes in patient- and carer-related measures (i.e., functional performance, quality of life, and carer preparedness) between T1 and T3, we calculated difference scores and 95% confidence interval (CI). Additionally, we conducted paired sample *t*-tests and Wilcoxon signed-rank tests to assess the statistical significance of these changes.

## Results

### Participant recruitment and eligibility

**Figure 2.**
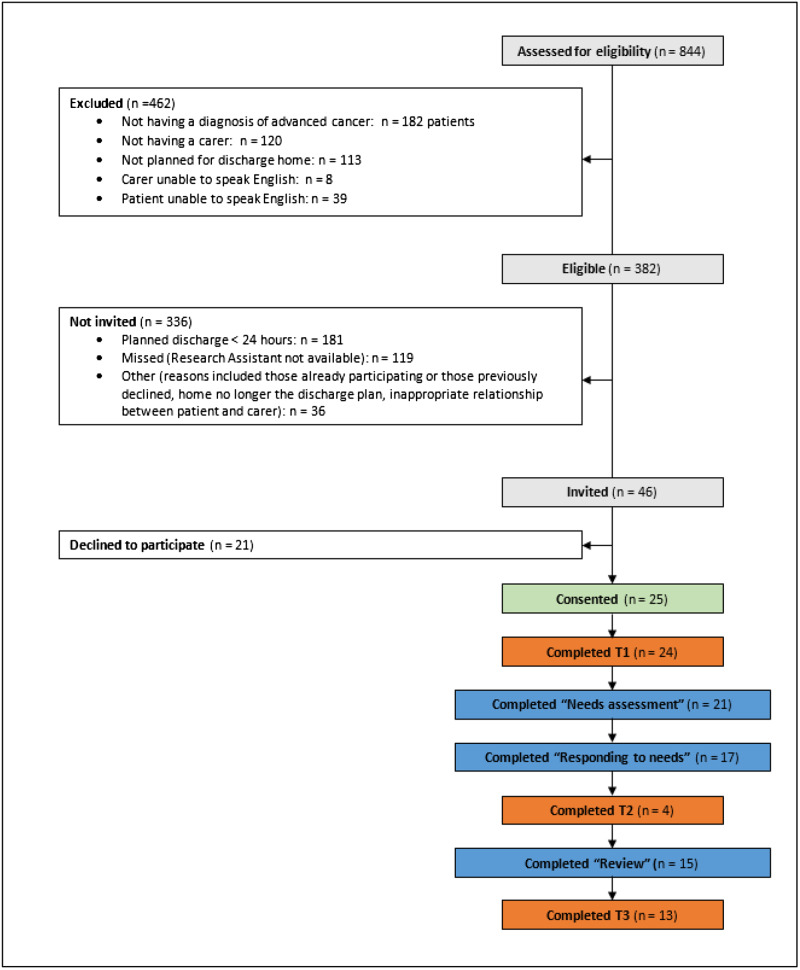
Recruitment and participation. T1 = timepoint 1 – 1 day before CARENET, T2 = timepoint 2 – 1–2 days before discharge, T3 = timepoint 3 – after CARENET and within 1 week after discharge.

Twenty-five dyads were recruited across the 8-month accrual period (Fig. 2). A high number (*n* = 336; 87%) of identified eligible participants were not invited to participate. Rapid and unpredicted hospital discharge was the primary reason (54%) for not inviting identified eligible participants, followed by the RA not being available (35%). There was a 54% consent rate for the remaining dyads. Figure 2 shows lower completion rates of patient- and carer-related measures at T2 (*n* = 4, 16%) compared to T1 (*n* = 24, 96%) and T3 (*n* = 13, 52%). Reasons included RA unable to contact the carer at time of discharge.

### Participant characteristics

Key characteristics of patient–carer dyads are shown in Tables 2 and 3. Carer participants had a mean age of 56.79 (SD = 15.54), were more likely to be female (56%, *n* = 14), were born in Australia (76%, *n* = 19), were English speaking at home (80%, *n* = 20), and were a spouse or partner to the person they were caring for (72%, *n* = 18). Seventy-two per cent (*n* = 18) lived with the person they cared for and 48% (*n* = 12) were still employed. Patient participants had a mean age of 59.72 (SD = 15.89), almost two-thirds (64%, *n* = 16) were female. Most patients spoke English at home (84%, *n* = 21), more than half were born in Australia (64%, *n* = 16), and 40% (*n* = 10) were still employed. There was high variance (heterogeneity) in the participant diagnosis, a median (IQR) length of stay in hospital of 17.0 (IQR = [14.5, 34.5]) days, and home was the discharge destination for 76% (*n* = 19) of patient participants.

**Table 2.** Demographic characteristics of consented carer participants (*n* = 25)

Carer participants		*n* (%)
Gender		
Male		11 (44)
Female		14 (56)
Age (years)		
Mean (SD)		56.79 (15.54)
Median (IQR)		57 (53.0, 71.0)
Missing (*n*)		6
Relationship to the patient		
Spouse/partner		18 (72)
(Adult) child		4 (16)
Other (e.g., parent, sibling, friend)		3 (12)
Level of education (completed?)		
Primary		0 (0)
Secondary		9 (36)
Tertiary		11 (44)
Missing		5 (20)
Employment status		
Employed		12 (48)
Unemployed		3 (12)
Retired		6 (24)
Missing		4 (16)
Primary language spoken at home		
English		20 (80)
Other		1 (4)
Missing		4 (16)
Country of birth		
Australia		19 (76)
Other		2 (8)
Missing		4 (16)
Length of time caring for the patient (months)		
Mean (SD)		14.52 (20.93)
Median (IQR)		5 (2.0, 16.0)
Missing (*n*)		4
Availability of carer		
Lives with patient		18 (72)
Lives with others		5 (20)
Missing		2 (8)
Other caregiving responsibilities		
Children, *n*		4
Spouse/partner, *n*		1
Other, *n*		5

**Table 3.** Demographic characteristics of consented patient participants (*n* = 25)

Patient participants		*n*(%)
Gender		
Male		9 (36)
Female		16 (64)
Age (years)		
Mean (SD)		59.72 (15.89)
Median (IQR)		63 (53.0, 72.5)
Level of education		
Primary		1 (4)
Secondary		10 (40)
Tertiary		8 (32)
Missing		6 (24)
Employment status		
Employed		10 (40)
Unemployed		3 (12)
Retired		8 (32)
Missing		4 (16)
Primary language spoken at home		
English		21 (84)
Other		3 (12)
Missing		1 (4)
Country of birth		
Australia		16 (64)
Other		9 (36)
Type of cancer		
Gynecological		5 (20)
Lung		4 (16)
Hematological		3 (12)
Lower gastro-intestinal		3 (12)
Skin and melanoma		3 (12)
Upper gastro-intestinal		2 (8)
Other^a^		5 (20)
Patient length of stay (days)		
Mean (SD)		23.84 (15.81)
Median (IQR)		17 (14.5, 34.5)
Number of readmissions within 30 days		
Mean (SD)		0.53 (0.61)
Median (IQR)		0 (0−2)
Missing (*n*)		6
Actual discharge destination		
Home		19 (76)
Passed away in hospital		5 (20)
Still in hospital at end of study		1 (4)

IQR: interquartile range.

a Other = Breast, head and neck, urological, cancer of unknown primary, neurological.

### Intervention adherence

Partial adherence was reached with 60% of enrolled participants completing all 3 intervention components (Fig. 2). Adherence to components occurring before discharge was higher than the review component occurring after discharge, with 84% of carer participants completing the needs assessment and 68% completing the needs assessment and creating an action plan to respond to needs. Reasons for non-adherence included patient death prior to completing the intervention (*n* = 5), patient discharge too early (*n* = 1), transfer to palliative care ward (*n* = 1), unable to contact carer (*n* = 2), and unknown (*n* = 1).

### Limited efficacy

Table 4 presents patient and carer outcomes (*n* = 12) assessed before (T1) and after (T3) the CARENET intervention. Upon returning home (T3), patients demonstrated a trend toward increased care needs compared to their hospital stay (T1), although they remained able to manage their own needs with limited physical assistance (AKPS mean [SD] = 68.89 [12.96] at T1 vs. 61.11 [24.21] at T3). Symptom burden also showed an upward trend upon returning home (SAS mean [SD] = 6.44 [4.56] at T1 vs. 11.11 [12.23] at T3).

**Table 4.** Patient and carer outcome measures taken before (T1) and after CARENET (T3) (*n* = 12)

Outcome	T1 mean (SD)	T3 mean (SD)	Difference (T1–T3)	95% CI
Preparedness for caregiving scale				
For physical care	2.50 (0.91)	2.83 (0.84)	−0.33	(−0.90, 0.23)
For emotional care	2.50 (1.01)	2.25 (0.87)	0.25	(−0.30, 0.80)
For service finding/setup	2.75 (0.87)	2.92 (0.79)	−0.17	(−0.70, 0.36)
For stress of caregiving	2.33 (1.07)	2.42 (0.10)	−0.08	(−0.82, 0.66)
To make caregiving pleasant	2.82 (0.75)	2.73 (0.79)	0.09	(−0.38, 0.56)
To handle emergencies	2.64 (1.03)	3.09 (0.83)	−0.46	(−1.27, 0.36)
To get support from the health system	2.75 (1.06)	3.17 (0.72)	−0.42	(−0.99, 0.16)
How prepared overall	2.75 (0.86)	3.25 (0.62)	−0.50	(−1.01, 0.01)
Total scores	21.09 (5.52)	22.36 (4.13)	−1.27	(−3.48, 0.94)
AKPS	68.89 (12.96)	61.11 (24.21)	7.78	(−5.41, 20.97)
RUG-ADL	4.44 (1.33)	7.33 (4.87)	−2.89	(−5.95, 0.17)
Symptom Assessment Scale	6.44 (4.56)	11.11 (12.23)	−4.67	(−12.15, 2.83)
Carer Quality of Life^a^	78.95 (14.09)	79.45 (12.45)	−10.47	(−17.10, −3.85)
EQ-5D-5L VAS self-assessed health rating (0−100)	49.11 (31.17)	59 (35.92)	−0.51	(−9.46, 8.44)

AKPS = Australian Karnofsky Performance Status, RUG-ADL = Resource Utilization Group – Activities of Daily Living, EQ-5D-5L = EuroQol 5-Level-5-Dimensions.

a 0–4 scale, response options 0 = not at all to 4 = very well prepared, MCID = 0.25 point difference per item or more than 2 point difference overall (REF).

Carers exhibited mixed outcomes, reporting a decreased sense of preparedness for addressing patients’ emotional needs (mean [SD] = 2.50 [1.01] at T1 vs. 2.25 [0.87] at T3) but improved preparedness for practical caregiving tasks, including obtaining system support (mean [SD] = 2.75 [1.06] at T1 vs. 3.17 [0.72] at T3), managing emergencies (mean [SD] = 2.64 [1.03] at T1 vs. 3.09 [0.83] at T3), finding services (mean [SD] = 2.75 [0.87] at T1 vs. 2.92 [0.79] at T3), and providing physical care (mean [SD] = 2.50 [0.91] at T1 vs. 2.83 [0.84] at T3). The Preparedness for Caregiving Scale revealed increases in mean scores across practical domains, although these changes were not statistically significant. Overall preparedness improved marginally from 2.75 (SD = 0.86) at T1 to 3.25 (SD = 0.62) at T3 (*p* = 0.05).

Notably, carers’ quality of life showed a significant improvement, increasing from 78.95 (SD = 14.09) before CARENET (T1) to 79.45 (SD = 12.45) after CARENET T3 (*p* = 0.01). While trends toward improvement were observed across several domains, including physical care, support from the health system, and overall preparedness, statistical significance was limited to carers’ quality of life and marginally to overall preparedness.

## Discussion

This was the first phase 2 trial to test the feasibility of delivering the CSNAT- I as an integrated part of discharge planning practices to assist with discharge from hospital to home for carers of people with advanced cancer. Our findings have shown that while the recruitment target was reached, there were several challenges to the feasibility of the intervention and study conduct.

First, a limited group of carers received the intervention, while a vast number of eligible carers did not participate. This is almost certainly due to a mismatch between the hospital discharge context and the trial’s eligibility procedures. Rapid discharge home for people with palliative care needs is commonplace in acute hospitals and can happen quickly, often within 24 h (Department of Health Victoria 2021; Australian Institute of Health and Welfare 2024). We found that for people planning to return home in 24 h there was insufficient time to complete all aspects of the intervention and study measures according to the protocol. Furthermore, the limited grant funding restricted the RA’s availability to 2 days per week and hindered the ability to reach all eligible participants. Another reason for smaller recruitment numbers could be clinician “gatekeeping” that excluded patient carers with perceived high levels of distress. Another CSNAT-I trial reported that clinicians who seek to avoid overwhelming carers may inadvertently limit support for those with greater needs (Lund et al. 2020).

Secondly, in this trial, delivery of all intervention components as intended was difficult, particularly the review after discharge. Recorded reasons for not completing all components (i.e., availability of carers and patient death) were like other trials delivering adapted CSNAT-I models (Toye et al. 2016; Patchwood et al. 2021). However, it is possible that problems with delivering the CARENET as planned may be because of the shortfalls in the implementation process and subsequent impact on clinicians’ proficiency. The recommended implementation and training strategy was used for the CSNAT-I component (The University of Manchester/ University of Cambridge 2022). However, this was impacted by high staff turnover, as well as reduced access to clinical champions, and the research staff for support and fidelity monitoring. Previous CSNAT-I studies recommend that these features are important for successful implementation in both trial and usual practice settings (Diffin et al. 2018; Hall et al. 2020).

It is also possible that difficulty with delivering CARENET could be attributed to using a protocolized, multi-staged delivery model that is not congruent with the fast-paced and dynamic nature of the hospital discharge context. Discharge home from hospital can occur along different pathways and timelines, compared to more stable and predictable community and home-based settings where most trials testing the CSNAT-I have previously occurred (Aoun et al. 2015; Grande et al. 2017; Lund et al. 2020).

Thirdly, the study showed that there were difficulties with the completion of the selected outcome measures. Obtaining data from patients and carers was difficult to achieve at discharge (T2), compared to before and after intervention (T1 and T3). The positive trends observed for carer quality of life and overall levels of preparedness after intervention align with findings of other CSNAT-I trials (Toye et al. 2016; Grande et al. 2017), supporting the use of these outcome measures to test the impact of carer-targeted interventions. However, results must be interpreted with caution, given the small sample size and the large group of carers deemed eligible but discharged prior to recruitment

## Implications and future directions

Our trial has highlighted the opportunities and the challenges of conducting carer-focused interventions in a compressed and unpredictable hospital discharge context. Foremost is the need to consider a study design that will increase access to a more diverse group of patient–carer dyads (i.e., age, ethnicity, and higher care needs) and ultimately improve the clinical translation of this type of intervention. This could involve using a more flexible, multi-modal eligibility process that allows recruitment to take place at various times and in settings that align with carers’ needs and their different pathways home (Gustavson et al. 2024). An example of this could be an opt-out “early” approach, where carers are automatically recruited to the study upon patient admission and have the choice to opt out. This opt-out “early” approach may also overcome barriers from limited resources and staff availability (Treweek et al. 2013)

A modifiable intervention model could also improve access for all carers who need support at discharge. This could entail intervening with carers at different levels with tailored delivery modes to accommodate their needs and circumstances. Research into carers of older hospitalized people supports flexible and adaptable models that offer single and multistage approaches and are considered feasible and effective (Levoy et al. 2022). For carers of hospitalized people with advanced cancer, further research is required to understand the degree of modifications that can occur without compromising the efficacy of the intervention itself (Levoy et al. 2022). This could include a review of what aspects of CARENET are considered core and those where flexibility of inclusion may apply. Tools to assist with adapting existing interventions are recommended to systematize this process and achieve a balance between access and impact (Kirk et al. 2020).

Stepped-care intervention models could offer a stratified approach where the level of care (or carer support) is matched to the level of complexity of the condition or individual need (General Practice Mental Health Standards Collaboration (GPMHS) 2017). Stepped care models are well established in mental health care and are used to optimize access and efficiencies within given resources (Berger et al. 2022; Mughal et al. 2023). Similarly, cancer prehabilitation models use a stepped care approach (MacMillan Cancer Support 2019) where all people having surgery are offered prehabilitation, but scope and intensity will vary. CARENET could similarly be implemented in this stepped-care approach, whereby intensity of support may differ according to need. For example, carers of people experiencing escalating carer needs would need a greater intensity of intervention to assist with discharge planning compared to carers of people whose care needs remain unchanged. If this approach were applied, additional feasibility studies would be needed to ensure intervention models are fit for purpose before proceeding to a larger scale study.

It can also be argued that understanding the impact of a person-centered intervention like CARENET should be informed by the carers’ engagement with the intervention, not just adherence to the intervention protocol. Assessing fidelity of enactment is considered a measure of engagement, specifically whether the intervention content leads to a change in behavior that is then enacted in daily life (Walton 2024). Even though consensus has not been reached in the literature regarding the best way to measure enactment, there is a shared opinion that high levels of enactment equate to good outcomes. Given that CARENET relies on carers responding to the needs assessment and the support offered for it to work, this aspect of fidelity should be considered in future feasibility study design alongside fidelity of delivery and receipt (Walton 2024).

Several limitations of this study should be noted. First, while the trial design testing feasibility is aligned with recommendations of the MRC framework (Skivington et al. 2021), the single-arm design with no comparable control group limited our scope to assess against usual care outcomes. Secondly, the generalizability of the findings to other carer groups and different hospital settings is limited. Thirdly, the small sample size limits the ability to comment upon the efficacy of CARENET on patient–carer outcomes. However, this was a feasibility trial where a pragmatic sample size was selected to focus on “limited efficacy” (Bowen et al. 2009) and help determine where the conduct of chosen outcome measures was practical and fit for scaling up for large efficacy trials.

## Conclusion

In conclusion, our trial supports the understanding that carers of people with advanced cancer or palliative care needs will continue to need significant support during the transition from hospital to home, no matter what the context. However, delivering a protocolized multi-staged intervention for carers in this discharge setting may have limited feasibility and compromise access to more support for all carers. Further exploration of what intervention content and delivery is needed in order to benefit all carers and the people they are caring for, before proceeding to larger-scale trials.

## Supporting information

10.1017/S1478951525100710.sm001 Marston et al. supplementary material Marston et al. supplementary material

## Supplementary material

The supplementary material for this article can be found at https://doi.org/10.1017/S1478951525100710.

## References

[ref1] Abernethy AP, Shelby-James T, Fazekas BS, et al. (2005) The Australia-modified Karnofsky Performance Status (AKPS) scale: A revised scale for contemporary palliative care clinical practice [ISRCTN81117481]. *BMC Palliative Care* 4, 1–12. 16283937 10.1186/1472-684X-4-7 PMC1308820

[ref2] Adejoh SO, Boele F, Akeju D, et al. (2021) The role, impact, and support of informal caregivers in the delivery of palliative care for patients with advanced cancer: A multi-country qualitative study. *Palliative Medicine* 35(3), 552–562. 33353484 10.1177/0269216320974925 PMC7975852

[ref3] Aoun S, Deas K, Toye C, et al. (2015) Supporting family caregivers to identify their own needs in end-of-life care: Qualitative findings from a stepped wedge cluster trial. *Palliative Medicine* 29(6), 508. 25645667 10.1177/0269216314566061

[ref4] Archbold PG, Stewart BJ, Greenlick MR, et al. (1990) Mutuality and preparedness as predictors of caregiver role strain. *Research in Nursing and Health* 13(6), 375–384. 2270302 10.1002/nur.4770130605

[ref5] Australian Institute of Health and Welfare (2024) Characteristics of palliative care-related hospitalisations. Available at https://www.aihw.gov.au/reports/palliative-care-services/palliative-care-services-in-australia/contents/admitted-patient-palliative-care/characteristics-of-palliative-care-related-hospital (accessed February 4 2025).

[ref6] Berger M, Fernando S, Churchill A, et al. (2022) Scoping review of stepped care interventions for mental health and substance use service delivery to youth and young adults. *Early Intervention in Psychiatry* 16(4), 327–341. 34018335 10.1111/eip.13180 PMC9292436

[ref7] Bowen DJ, Kreuter M, Spring B, et al. (2009) How we design feasibility studies. *American Journal of Preventive Medicine* 36(5), 452–457. 19362699 10.1016/j.amepre.2009.02.002 PMC2859314

[ref8] Carers UK(2021) Carers experiences of hospital discharge - discharge to assess model. Available at https://www.carersuk.org/for-professionals/policy/policy (accessed January 31 2025). 722 library/carer-s-experiences-of-hospital-discharge-discharge-to-assess-model (accessed January 31 2025)

[ref9] Courvoisier DS, Aoun SM, Grande G, et al. (2015) The Impact of the Carer Support Needs Assessment Tool (CSNAT) in community palliative care using a stepped wedge cluster trial. *PLoS One* 10(4), 1–16. doi:10.1371/journal.pone.0123012 PMC4388632 25849348

[ref10] Daveson BA, Allingham SF, Clapham S, et al. (2021) The PCOC Symptom Assessment Scale (SAS): A valid measure for daily use at point of care and in palliative care programs. *PLoS One* 16(3), e0247250. 33765077 10.1371/journal.pone.0247250 PMC7993777

[ref11] Department of Health Victoria (2021) Discharge planning at end of life. Available at https://www.health.vic.gov.au/patient-care/discharge-planning-at-end-of-life (accessed 2 February 2025).

[ref12] Diffin J, Ewing G, Harvey G, et al. (2018) The Influence of Context and Practitioner Attitudes on Implementation of Person- Centered Assessment and Support for Family Carers Within Palliative Care. *World Views on Evidence Based Nursing* 15(5), 377–385. 10.1111/wvn.12323 30152150

[ref13] Eldridge SM, Chan CL, Campbell MJ, et al. (2016) CONSORT 2010 statement: Extension to randomised pilot and feasibility trials. *BMJ* 355, 1–29. 10.1136/bmj.i5239 PMC5076380 27777223

[ref14] Eva G and Morgan DM (2018) Mapping the scope of occupational therapy practice in palliative care: A European Association for Palliative Care cross-sectional survey. *Palliative Medicine* 32(5), 960–968. 29756556 10.1177/0269216318758928 PMC5946674

[ref15] Ewing G, Brundle C, Payne S, et al. (2013) The Carer Support Needs Assessment Tool (CSNAT) for use in palliative and end-of-life care at home: A validation study. *Journal of Pain and Symptom Management* 46(3), 395–405. 23245452 10.1016/j.jpainsymman.2012.09.008

[ref16] Ewing G AL, Jones D and Grande G (2018) Who cares for the carers at hospital discharge at the end of life? A qualitative study of current practice in discharge planning and the potential value of using The Carer Support Needs Assessment Tool (CSNAT) Approach. *Palliative Medicine* 32(5), 939–949. 29490198 10.1177/0269216318756259 PMC5946661

[ref17] Fries BE, Schneider DP, Foley WJ, et al. (1994) Refining a case-mix measure for nursing homes: Resource Utilization Groups (RUG-III). *Medical Care* 32(7), 668–685. 8028403 10.1097/00005650-199407000-00002

[ref18] General Practice Mental Health Standards Collaboration (GPMHS) (2017) System change towards a Stepped Care Model. Available at https://gpmhsc.org.au/guidelines/index/9f6d69d0-8660-4520-afd8-93b50ac47243 (accessed 2 February 2025).

[ref19] Grande GE, Austin L, Ewing G, et al. (2017) Assessing the impact of a Carer Support Needs Assessment Tool (CSNAT) intervention in palliative home care: A stepped wedge cluster trial. *BMJ Supportive & Palliative Care* 7(3), 326–334. 10.1136/bmjspcare-2014-000829 PMC5574387 26719349

[ref20] Gustavson AM, Horstman MJ, Cogswell JA, et al. (2024) Caregiver recruitment strategies for interventions designed to optimize transitions from hospital to home: Lessons from a randomized trial. *Trials* 25(1), 454. 38965624 10.1186/s13063-024-08288-2 PMC11223294

[ref21] Hall A, Ewing G, Rowland C, et al. (2020) A drive for structure: A longitudinal qualitative study of the implementation of the Carer Support Needs Assessment Tool (CSNAT) intervention during hospital discharge at end of life. *Palliative Medicine* 34(8), 1088–1096. 32491967 10.1177/0269216320930935 PMC7388143

[ref22] Harding R, List S, Epiphaniou E, et al. (2012) How can informal caregivers in cancer and palliative care be supported? An updated systematic literature review of interventions and their effectiveness. *Palliative Medicine* 26(1), 7–22. doi:10.1177/0269216311409613 21737481

[ref23] Herdman M, Gudex C, Lloyd A, et al. (2011) Development and preliminary testing of the new five-level version of EQ-5D (EQ-5D-5L). *Quality of Life Research* 20, 1727–1736. 21479777 10.1007/s11136-011-9903-x PMC3220807

[ref24] Higginson IJ, Sarmento VP, Calanzani N, et al. (2013) Dying at home – is it better: A narrative appraisal of the state of the science. *Palliative Medicine* 27(10), 918–924. doi:10.1177/0269216313487940 23698451

[ref25] Hoefman RJ, van Exel J and Brouwer WB (2017) Measuring care-related quality of life of caregivers for use in economic evaluations: CarerQol tariffs for Australia, Germany, Sweden, UK, and US. *Pharmacoeconomics* 35, 469–478. 28039617 10.1007/s40273-016-0477-x PMC5357482

[ref26] Kirk MA, Moore JE, Wiltsey Stirman S, et al. (2020) Towards a comprehensive model for understanding adaptations’ impact: The model for adaptation design and impact (MADI). *Implementation Science* 15, 1–15. 32690104 10.1186/s13012-020-01021-y PMC7370455

[ref27] Klaic M, Kapp S, Hudson P, et al. (2022) Implementability of healthcare interventions: An overview of reviews and development of a conceptual framework. *Implementation Science* 17(1), 10. 35086538 10.1186/s13012-021-01171-7 PMC8793098

[ref28] Lage DE, Nipp RD, D’Arpino SM, et al. (2018). Predictors of posthospital transitions of care in patients with advanced cancer. *Journal of Clinical Oncology* 36(1), 76–82. 29068784 10.1200/JCO.2017.74.0340 PMC5756321

[ref29] Laugaland K, Aase K and Barach P (2012) Interventions to improve patient safety in transitional care – A review of the evidence. *Work* 41, 2915–2924. doi:10.3233/wor-2012-0544-2915 22317162

[ref30] Lawson B, Burge FI, Critchley P, et al. (2006) Factors associated with multiple transitions in care during the end of life following enrollment in a comprehensive palliative care program. *BMC Palliative Care* 5(1), 1–10. doi:10.1186/1472-684X-5-4 PMC1557663 16734892

[ref31] Levoy K, Rivera E, McHugh M, et al. (2022) Caregiver engagement enhances outcomes among randomized control trials of transitional care interventions: A systematic review and meta-analysis. *Medical Care* 60(7), 519–529. 35679175 10.1097/MLR.0000000000001728 PMC9202479

[ref33] Lund L, Ross L, Petersen MA, et al. (2020) Effect of the Carer Support Needs Assessment Tool intervention (CSNAT-I) in the Danish specialised palliative care setting: A stepped- wedge cluster randomised controlled trial. *BMJ Supportive & Palliative Care* **14**(e), e772–e783. 10.1136/bmjspcare-2020-002467 33115831

[ref34] MacMillan Cancer Support (2019) Principles and guidance for prehabilitation within the management and support of people with cancer. Royal College of Anaesthetists and the National Institute for Health Research Cancer and Nutrition Collaboration. Available at https://cdn.macmillan.org.uk/dfsmedia/1a6f23537f7f4519bb0cf14c45b2a629/1532-10061/prehabilitation-for-people-with-cancer-tcm9-353994 (accessed 2 February 2025).

[ref35] Marston C, Agar M and Brown T (2015) Patients’ and caregivers’ perceptions of occupational therapy and adapting to discharge home from an inpatient palliative care setting. *British Journal of Occupational Therapy* 78(11), 688–696.

[ref36] Marston C, Morgan DD, Philip J, et al. (2022) Supporting carers as patients move between hospital and home: A systematic review of interventions to support these transitions in care. *Journal of Palliative Medicine* *26*(2), 270–298. 10.1089/jpm.2022.0221 36251853

[ref37] Marston C, Morgan DD, Philip J, et al. (2023) Experience and acceptability of a carer‐focussed intervention in acute oncology settings: A qualitative study of people with advanced cancer and their carers. *Australian Occupational Therapy Journal* 70(5), 570–580. 37271728 10.1111/1440-1630.12887

[ref38] Mughal S, Salmon A, Churchill A, et al. (2023) Guiding principles for implementing stepped care in mental health: Alignment on the bigger picture. *Community Mental Health Journal* 59(6), 1035–1042. 37002486 10.1007/s10597-023-01116-y

[ref39] National Institute of Health and Clinical Excellence (NICE) (2004) Guidance on cancer services. Improving supportive and palliative care for adults with cancer. The manual. Great Britain. Available at https://www.nice.org.uk/guidance/csg4 (accessed 2 February 2025).

[ref40] Naylor MD, Shaid EC, Carpenter D, et al. (2017) Components of comprehensive and effective transitional care. *Journal of the American Geriatrics Society* 65(6), 1119–1125. 28369722 10.1111/jgs.14782 PMC5497308

[ref41] Patchwood E, Woodward-Nutt K, Rhodes SA, et al. (2021) Organising Support for Carers of Stroke Survivors (OSCARSS): A cluster randomised controlled trial with economic evaluation. *BMJ Open* 11(1), e038777. 10.1136/bmjopen-2020-038777 PMC7805348 33436463

[ref42] Skivington K, Matthews L, Simpson SA, et al. (2021) A new framework for developing and evaluating complex interventions: Update of Medical Research Council guidance. *BMJ* 374, 1–11. 10.1136/bmj.n2061 PMC8482308 34593508

[ref43] Totton N, Lin J, Julious S, et al. (2023) A review of sample sizes for UK pilot and feasibility studies on the ISRCTN registry from 2013 to 2020. *Pilot and Feasibility Studies* 9(1), 188. 37990337 10.1186/s40814-023-01416-w PMC10662929

[ref44] Toye C, Parson R, Slatyer S, et al. (2016) Outcomes for family carers of a nurse-delivered hospital discharge intervention for older people (the Further Enabling Care at Home Program): Single blind randomised controlled trial. *International Journal of Nursing Studies* 64, 32–41. 27684320 10.1016/j.ijnurstu.2016.09.012

[ref45] Treweek S, Lockhart P, Pitkethly M, et al. (2013) Methods to improve recruitment to randomised controlled trials: Cochrane systematic review and meta-analysis. *BMJ Open* 3(2), e002360. doi:10.1136/bmjopen-2012-002360 PMC3586125 23396504

[ref46] The University of Manchester/ University of Cambridge (2022) CSNAT-I Training and Implementation Toolkit. Available at https://csnat.org/training-for-use-in-practice/ (accessed 2 February 2025).

[ref47] Walton H (2024) *Towards Comprehensive Fidelity Evaluations: Consideration of Enactment Measures in Quality Improvement Interventions*. BMJ Publishing Group Ltd: American Society of Clinical Oncology. 10.1136/bmjqs-2023-016592 37714701

[ref48] Wang T, Molassiotis A, Chung BPM, et al. (2018) Unmet care needs of advanced cancer patients and their informal caregivers: a systematic review. *BMC Palliat Care* 17(1), 96. doi:10.1186/s12904-018-0346-9 30037346 PMC6057056

[ref49] Whitney RL, Bell JF, Tancredi DJ, et al. (2017) Hospitalization rates and predictors of rehospitalization among individuals with advanced cancer in the year after diagnosis. *Journal of Clinical Oncology* 35(31), 3610–3617. 28850290 10.1200/JCO.2017.72.4963 PMC5946701

[ref50] World Health Organization (WHO) (2016) Transitions of care. 9241511591. Available at https://iris.who.int/bitstream/handle/10665/252272/9789241511599-eng.pdf (accessed 2 February 2025).

