# Supplementary material for: Caring for carers of people with advanced cancer at hospital discharge (CARENET): A single-arm open label feasibility trial
Source: Palliat Support Care. 2025 Sep 5;23:e156. doi: 10.1017/S1478951525100710 (PMC13166733; doi:10.1017/S1478951525100710)
Supplement: Marston et al. supplementary material [file S1478951525100710sup001.zip › S1478951525100710sup001/Supplementary File 1.docx]

Supplementary File 1: CARENET Intervention

| **What:** Materials: The 5-staged licensed Carer Support Needs Assessment Tool - Intervention (CSNAT-I) was used (Ewing et al 2013). It consisted of a validated paper-based form that included 15 domains of need, criteria to rate and prioritise needs, and a template to outline action plans to address needs (csnat.org). Domains fell into two categories – Support domains to enable the carer to care (e.g. “Do you need more support with understanding your relative’s illness?”) and support domains for carers themselves (e.g., “Do you need more support with having time for yourself in the day?”) This paper-based form was a record that carers kept and clinicians’ documented summary in the electronic medical record. Online learning modules were used to train intervention providers and were accessible via the website once license was approved (csnat.org). A local protocol was developed and used to assist with intervention delivery and documentation into electronic medical record.  Procedures: The intervention included three components: (1) Needs Assessment - identification and prioritisation of needs, (2) Responding to needs - development of action plan for prioritised needs and (3) Review of whether needs have been addressed, and structured handover of outstanding needs. Specific stages of the CSNAT-I were embedded within these components and is described below.  *Needs Assessment* After the Occupational Therapist (OT) completed an initial assessment with patients to determine their needs related to achieving discharge home (e.g., functional limitations, physical care needs, Quality of life (QOL) goals); they met with the carer to determine their discharge needs using the CSNAT-I paper based tool (Stages 1-3). This occurred face to face, via telehealth or the phone depending on carer preference and access. The assessment process involved providing carers with the 14 domains of need, carers self-rating how much support they thought they needed and describing how the need relates to them. For example, the domain “needing help with equipment” may relate to a carer wanting to know how to use a manual lifting device. The carer then chose three needs they described that they wished to prioritise and seek support with before discharge home. *Responding to needs:* The OT identified what actions were required to address prioritised needs with the carer (Following Stage 4 of CSNAT-I). The OT and carer then determined together which actions needed to be communicated to the patient and team. Actions included direct OT interventions, referrals to other disciplines and providers; or carer-led initiatives. These were documented on the CSNAT-I and copies kept by both the carer and OT. The OT actioned the relevant items. A pre-discharge review occurred if needed by the carer e.g., to check they had all the equipment delivered to their house. Proposed and enacted action plans were communicated to the patient and team members as required. *Review:* Prior to discharge the OT asked the carer to nominate the preferred method and time of review required after discharge. Options were: (1) treating OT to phone at time proposed by carer; (2) another key contact chosen by the carer (social worker, nurse coordinator) will contact carer instead of the OT, or (3) carer contacts treating OT or key contact person at preferred time. Shared review (Stage 5 CSNAT-I) involved (1) checking if the action plan had been completed, and (2) addressing outstanding or new needs identified by, or referring to, relevant service providers (e.g., GP, community palliative care service, nurse coordinators).  In addition to the CSNAT-I, usual discharge planning processes were followed and tailored to the patient and the carer. |
| --- |
| **Who:** Trained occupational therapists facilitated the delivery of the intervention. Multi-disciplinary interventions were delivered (in response to CSNAT-I) and based on individual need |
| **How:** Components 1 and 2 were delivered either face-to-face, telehealth or via phone; component 3 was delivered via telehealth or via phone and involved the OT and the carer for the patient. |
| **Where:** Components 1 and 2 occurred during acute admission and component 3 occurred once the patient was discharged from the acute hospital. Treating occupational therapists delivering the intervention were hospital-based. |
| **When and how much:** Components 1 and 2 were delivered during hospital stay and when discharge home had been decided. Component 3 was delivered within 72 hours after discharge (or other times nominated by carer, but no more than 1 week after discharge). Components 1 and 2 occurred as separate or combined contacts between the OT and carer. It was anticipated that these components would take between 20-40 minutes; and component 3 between 5 -20 minutes. However, this depended on individual needs and whether it occurred within other bundles of care e.g. psychoeducation. If the person with advanced cancer had a change in planned discharge destination, anticipated care needs, length of stay in hospital; different stages of the intervention could be repeated  Tailoring A person-centred model allowed for the intervention delivery process, the actions and care generated to be adapted to the individual carer and their own preferences. |
