# Supplementary material for: Caring for carers of people with advanced cancer at hospital discharge (CARENET): A single-arm open label feasibility trial
Source: Palliat Support Care. 2025 Sep 5;23:e156. doi: 10.1017/S1478951525100710 (PMC13166733; doi:10.1017/S1478951525100710)
Supplement: Marston et al. supplementary material [file S1478951525100710sup001.zip › S1478951525100710sup001/Supplementary File 2 Summary of measures.docx]

Supplementary File 2 Summary of measures

| Name | Author | Purpose | Subscales | Items | Item scores | Range | Validity and Reliability |
| --- | --- | --- | --- | --- | --- | --- | --- |
| Preparedness for Caregiving Scale | Archbold et al (1990) | Shows change over time in caregiving preparedness (3 time points). MCID* is 2 points or more in total score or more than 0.25 points per item (REF). | None | 8 | 0=not at all to 4=very well prepared | 0-32 | Satisfactory internal consistency, reliability and stability, and one-dimensionality (Archbold et all, 1990, Henriksson er al, 2012) |
| Australia-Modified Karnofsky Performance Scale (AKPS) | Abernethy et al (2005) | Provides an indication of the patient’s overall performance status. | None | 1 | 0=dead to 100=normal; no complaints; no evidence of disease | 0-100 | High inter-rater reliability and is sensitive to changes over time (Abernathy et al, 2005). |
| Resource Utilisation Groups – Activities of Daily Living (RUG – ADL) | Fries et al (1994) | Measures patients’ functional performance according to levels of assistance and resources needed when completing activities of daily living. | None | 4 | Bed mobility, toileting, transfer (1=independent/supervision to 5=two-person assist); eating (1=independent/supervision to 3=extensive assistance/total dependence/tube feed) | 4-18 | High inter-rater reliability and good reliability (Bjorkgren, 1999). |
| EuroQol 5-Dimensions-5-Levels | Herdman et al (2011) | Measurement of health-related quality of life. | VAS Health | 6 | Mobility,self-care, usual activities, pain/discomfort, anxiety/depression (1=no problem to 5=extreme problems/unable to); VAS health (0=worst health you can imagine to 100=best health you can imagine). | Mobility, self-care, usual activities, pain/discomfort, anxiety/depression (0-25); VAS Health (0-100) | Demonstrated validity in health settings (Fermont, 2017). |
| Carer Quality of Life (QOL) Instrument | Hoefman et al (2013) | Shows the quality of life of carers and the subjective burden of caring. | CarerQOL VAS | 8 | No=1 to A lot of=3; CarerQOL VAS (0=completely unhappy to 10=completely happy). | 7-21; Carer QOL VAS (0-10) | Valid measure for carer research & economic evaluations (Hoefman et al, 2013) and (Voormolen et al, 2021) |
| Symptom Assessment Scale (SAS) | Daveson et al 2021 | A measurement of distress in the most common palliative care symptoms. | No | 7 | 0=Absent to 10=Severe. | 0-70 | Valid and reliable across palliative care settings (Daveson et al 2021). |

*MCID = minimal clinically important difference
