# Supplementary material for: Caring for carers of people with advanced cancer at hospital discharge (CARENET): A single-arm open label feasibility trial
Source: Palliat Support Care. 2025 Sep 5;23:e156. doi: 10.1017/S1478951525100710 (PMC13166733; doi:10.1017/S1478951525100710)
Supplement: Marston et al. supplementary material [file S1478951525100710sup001.zip › S1478951525100710sup001/Supplementary File 3.docx]

**Supplementary File 3: Implementation Strategy**

- Based on data from earlier acceptability study, clinician education and training occurred in the following ways:
- Individual training of the CSNAT-I using evidence based online learning (1 month before data collection starts)
- Group training on CARENET intervention protocol (2 weeks before recruitment starts)
- Assigned clinical champions to lead working group and 1:1 support for intervention delivery. Frequency of this group was determined by need.
- Training for all treating staff occurred via online modules, and the research team provided local training of adapted delivery protocol and trial processes.
- A working group of the treating OTs, consumers and researchers was established to optimise alignment to protocol and provide opportunity to address issues with fidelity.
- Education and training took place 1 month before data collection
- A simple study operational manual was developed
- The primary investigator and the RA attended the site and promote fidelity to study protocol, assisted with training and education and address any issues impacting on intervention delivery and study conduct.
